# Interactions between a pollinating seed predator and its host plant: the role of environmental context within a population

**DOI:** 10.1002/ece3.1134

**Published:** 2014-06-22

**Authors:** Abigail A R Kula, Dean M Castillo, Michele R Dudash, Charles B Fenster

**Affiliations:** 1 Department of Biology, University of MarylandCollege Park, Maryland, 20742; 2 Mountain Lake Biological StationPembroke, Virginia, 24136; 3 Department of Biology, Indiana UniversityBloomington, Indiana, 47405

**Keywords:** *Hadena*, oviposition, plant–insect interactions, pollination, *Silene*

## Abstract

Plant–insect interactions often are important for plant reproduction, but the outcome of these interactions may vary with environmental context. Pollinating seed predators have positive and negative effects on host plant reproduction, and the interaction outcome is predicted to vary with density or abundance of the partners. We studied the interaction between *Silene stellata*, an herbaceous perennial, and *Hadena ectypa*, its specialized pollinating seed predator. *Silene stellata* is only facultatively dependent upon *H. ectypa* for pollination because other nocturnal moth co-pollinators are equally effective at pollen transfer. We hypothesized that for plants without conspecific neighbors, *H. ectypa* would have higher visitation rates compared to co-pollinators, and the plants would experience lower levels of *H. ectypa* pollen deposition. We predicted similar oviposition throughout the study site but greater *H. ectypa* predation in the area without conspecific neighbors compared to plants embedded in a naturally high density area. We found that *H. ectypa* had consistently higher visitation than moth co-pollinators in all host plant contexts. However, *H. ectypa* pollinator importance declined in areas with low conspecific density because of reduced pollen deposition, resulting in lower seed set. Conversely, oviposition was similar across the study site independent of host plant density. Greater likelihood of very high fruit predation combined with lower pollination by *H. ectypa* resulted in reduced *S. stellata* female reproductive success in areas with low conspecific density. Our results demonstrate local context dependency of the outcomes of pollinating seed predator interactions with conspecific host plant density within a population.

## Introduction

Outcomes of interspecific interactions may shift along a continuum from mutualistic to antagonistic, and the context within which the interaction occurs may affect the placement of the interaction along the continuum (Bronstein 1994). Examining the effect of context on the outcome of interspecific interactions has been recognized as an important area of research for population and community ecology (Agrawal et al. 2007), and one such context is the varying abundance or density of one or both participants (Bronstein 1994; Holland and DeAngelis 2009).

Patches of plants that are small or have low conspecific density frequently occur in nature (e.g., during establishment of new populations within or outside the species range or after plant population fragmentation via habitat destruction), and the persistence of these patches is dependent on the successful reproduction of resident plants (Saunders et al. 1991; Debinski and Holt 2000; Groom 2001). However, the outcomes of interspecific interactions that affect plant reproduction (e.g., pollination and herbivory) may be context dependent and affected by host plant density (Root 1973; Kunin 1997; Kearns et al. 1998; Aguilar et al. 2006). Plants in small patches often experience reduced pollinator visitation and pollen deposition rates (Cunningham 2000; Aguilar et al. 2006; Andrieu et al. 2009; although specialized insect pollinators may utilize specific attractants to locate their host plants, Raguso 2008). Alternatively, when there is low host plant density, levels of herbivory may increase (Fagan et al. 2005; Gunton and Kunin 2009) or decrease (Kery et al. 2001) resulting in more or less negative effects on plant reproduction compared to the effects at higher density. A few studies simultaneously examine both pollination and herbivory in low or high density patches and show similar results for pollination and herbivory or seed predation interactions (Jules and Rathcke 1999; Groom 2001; Metcalfe and Kunin 2006; Garcia and Chacoff 2007). Thus, these studies demonstrate that the combination of beneficial and detrimental interspecific interactions could result in an overall change in the outcome of plant–insect interactions for host plant reproduction based on population context.

In their interactions with host plants, pollinating seed predators are unique in that they fulfill dual roles as both pollinators and seed predators. Adult insects provide pollination services for the host plant, but females also lay eggs on or in the flowers. The pollinator's offspring subsequently consume seeds and/or one or multiple fruits. These interactions range from obligate to facultative. In obligate interactions, both host plant and insect species are dependent upon each other (e.g., figs and fig wasps, Wiebes 1979). With facultative interactions, host plants receive additional pollination services from co-pollinators that do not consume seeds, but usually the pollinating seed predator species are obligately dependent on the host plant (e.g., *Silene* and *Hadena*, Kephart et al. 2006). The outcomes of interactions that are facultative for the host plant are predicted to be more variable among populations and therefore provide opportunities to explore ecological contexts in which pollinating seed predators are mutualistic versus parasitic to host plant reproduction (Dufay and Anstett 2003). By understanding where or when the interactions are mutualistic, we may be able to predict the ecological conditions that result in their evolution and persistence despite the presence of co-pollinator mutualists (Kephart et al. 2006; Bernasconi et al. 2009).

Other studies of pollinating seed predators and their host plants have found variable outcomes (in terms of host plant reproductive success) with differences in plant or insect abundance or density (Elzinga et al. 2005; Despres et al. 2007; Klank et al. 2010; Reynolds et al. 2012) indicating that density may play a role in their context dependency. However, none of these prior studies examined the effect of host plant density in terms of pollination efficiency of a pollinating seed predator. By studying the effect of host plant conspecific neighbor density on pollination efficiency, in particular, we will gain a mechanistic understanding of context-dependent outcomes of facultative pollinating seed predator interactions.

We quantified the interaction outcomes between a specialized pollinating seed predator, *Hadena ectypa* (Noctuidae)*,* and its host plant, *Silene stellata* (Caryophyllaceae), within areas that differed by host plant density. *Silene stellata* is facultatively dependent upon *H. ectypa* for pollination due to the presence of generalist and equally effective co-pollinators (Reynolds et al. 2012). We made the following four predictions. First, *H. ectypa* visitation would be similar among high and low density areas and higher than co-pollinator visitation in the context of low conspecific plant density because of specific *S. stellata* scents that are likely attractive to *H. ectypa* (Castillo et al. 2014). Second, reduced pollen movement by *H. ectypa* from the area with high conspecific host plant density to low density plants would result in lower seed set for low density host plants. Third, *H. ectypa* oviposition would be similar among the areas because visitation and oviposition are correlated (Reynolds et al. 2012). Finally, despite similar oviposition among areas, *H. ectypa* predation on low density plants would be higher because without neighboring plants, there is low flower and fruit availability for *H. ectypa* larvae, which therefore would be forced to consume more fruits per (experimental) host plant. From these specific predictions based on individual facets of the interaction, *H. ectypa* is predicted to be more antagonistic for *S. stellata* at low versus high density context because of the combination of reduced effectiveness as an adult pollinator and increased larval flower and fruit predation.

## Methods

### Study organisms

*Silene stellata* is a long-lived, iteroparous herbaceous plant that occurs in open meadows or closed canopy woods across the eastern half of the United States. Flowers are self-compatible and protandrous, and approximately 30 mm in diameter with fringed, white petals. Ovaries contain a mean of 25 ovules (Reynolds et al. 2009). In our study site, individual plants with one to several stems produce a mean ± SE of 75 ± 5 flowers, and our mainly single-stemmed study plants produced a mean ± SE of 24 ± 4 flowers. Flowering frequently begins in early July at our study site and ceases in early September. *Silene stellata* exhibits the typical nocturnal moth pollination syndrome: anther dehiscence, stigma receptivity, and scent production occur at dusk, and indeed, nocturnal moths are the primary pollinators. Plants are also able to set low levels of seed autonomously when enclosed in cages that prevent insect visitation (resulting in no pollinator-mediated pollen exchange) (Reynolds et al. 2009).

Of the moth pollinator community, *Hadena ectypa* is a specialized pollinator and seed predator of *S. stellata*. After feeding on nectar, female *H. ectypa* may oviposit (∼40% of all *H. ectypa* visits), most typically at the base of the ovary inside the large and persistent calyx; moths that feed on nectar and then oviposit do not deposit more pollen on stigmas compared to moths that only feed on nectar and do not oviposit (Kula et al. 2013). Eggs adhere to the ovary or inside calyx wall and can be reliably counted after fruit collection from the field. The larvae feed on flowers and fruits, each larva consuming approximately 30–40 flowers or fruits in the lab before pupation (Reynolds et al. 2012), and larvae prefer younger, softer fruits (Castillo et al. 2013). In almost all cases, the flower or fruit is completely consumed with no ovules or seeds remaining. Moth co-pollinators in three families (Arctiidae, Noctuidae and Notodontidae) pollinate but do not oviposit on *S. stellata* flowers (Reynolds et al. 2009), and their relative abundance compared to *H. ectypa* varies annually and within the flowering season (Kula 2012; Reynolds et al. 2012). Because *H. ectypa* and moth co-pollinators are similarly effective at *S. stellata* pollen transfer (Reynolds et al. 2012; Kula et al. 2013), abundance data alone may be used to infer their importance as pollinators of *S. stellata* (Reynolds et al. 2012), where importance reflects the product of frequency and efficiency of pollinators (Reynolds and Fenster 2008).

### Study area

**Figure 1:**
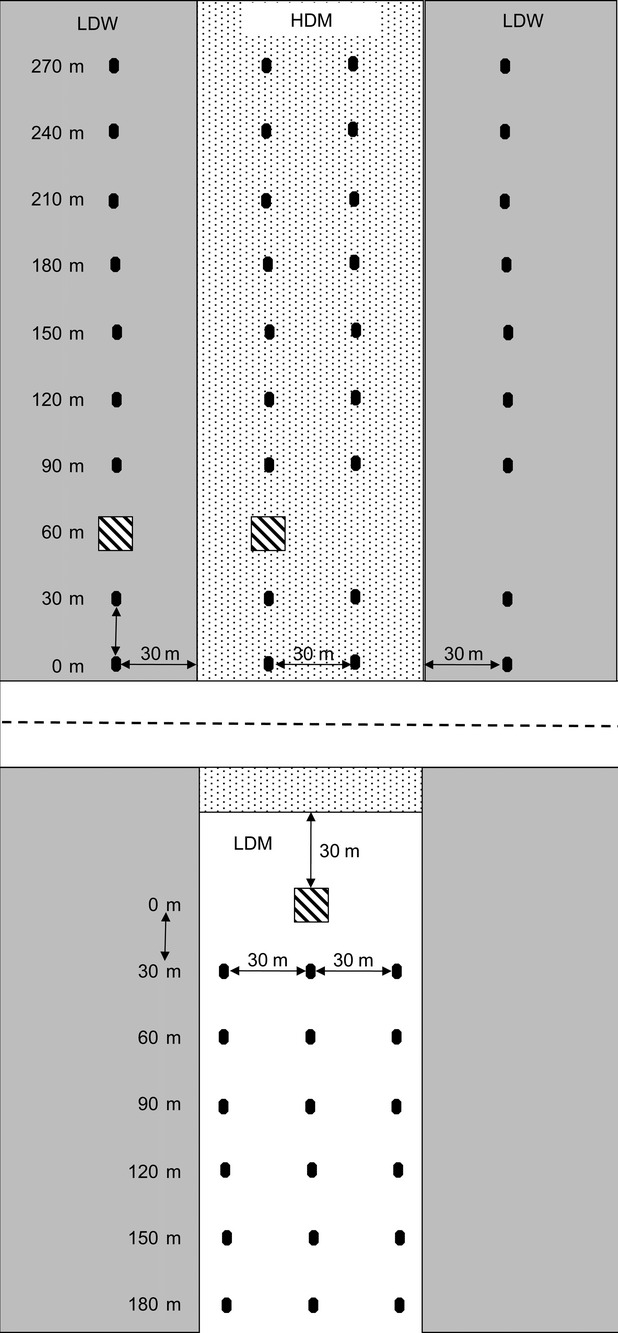
Schematic diagram of *Silene stellata* experimental transects in naturally occurring high density (HDM) field population and manipulated low density woods (LDW) and low density meadow (LDM) areas. Gray-filled areas on the sides of the diagram represent wooded habitat, while the center area is an open meadow habitat within a power line cut. The stippled area at the top of the diagram specifically represents the only area with a continuous population of naturally occurring *S. stellata* plants. Squares with diagonal stripes represent the approximate location of patches of potted plants placed in the field for pollinator observations in 2008 and 2009 and for flower collection for stigmatic pollen loads in 2008. Closed circles represent the approximate placement of individual potted plants used for fruit set, seed set, oviposition, and predation data collection in 2009.

**Figure 2:**
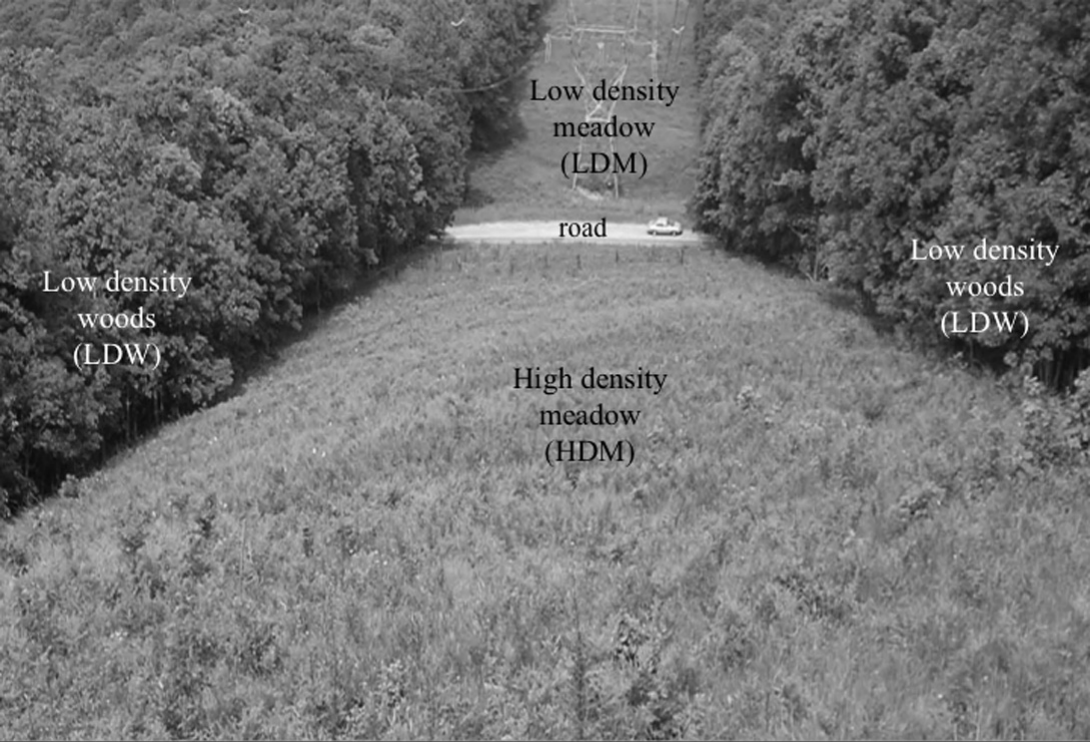
Photograph of the field site taken from the highest point within the site. A road bisects the meadow area, and the high density meadow (HDM) with an abundant population of naturally occurring *Silene stellata* is shown at the bottom of the photo. The low density woods (LDW) areas occur on either side of the high density meadow. The low density meadow (LDM) occurs at the top of the photo and up the mountainside to the road.

Our study was conducted near Mountain Lake Biological Station (Giles County, Virginia, USA) within and adjacent to a large, continuous population of *S. stellata* that naturally occurs in a meadow within a power line cut (∼45 m wide; 37°20′53″N, 80°32′41″W, elevation ≈ 1100–1300 m) where density is approximately 1.5 plants per m^2^, which is at the highest end of natural *S. stellata* population densities (personal observations). For our study, this meadow is the high density meadow (hereafter referred to as HDM) area (Figs. 1, 2).

This is the only population of suitable size and nighttime accessibility within 10 km, dictating an experimental approach to test our hypotheses. Because we were not able to uniformly mow or reduce plant density within this population, we chose to manipulate host density by taking advantage of adjacent areas not currently (or within the last 10 years) colonized by *S. stellata*. Using areas adjacent to an established *S. stellata* population was also important because in order to test predictions of host-specific pollinating seed predator moth visitation rates, our study areas needed to be within the expected flight capabilities of moths from an established population (see more below on spacing).

Our two low density areas were located adjacent to the high density meadow (Figs. 1, 2). The low density woods (hereafter referred to as LDW) was closed canopy woodland adjacent to both sides of the high density meadow, and the habitat is similar to wooded habitat nearby where *S. stellata* is established (Reynolds et al. 2012). The low density meadow (hereafter referred to as LDM) area was below a paved road that perpendicularly bisects the same power line cut but within a large area where *S. stellata* does not occur.

In all three areas across this study site, *S. stellata* was the only nocturnally flowering species present during our study. Mean light level in the LDW was 4% of the mean light level in the HDM [HDM: 3516.9 *μ*mol·s^−1^·m^−2^, LDW: 132.6 *μ*mol·s^−1^·m^−2^,; as measured in the afternoon under partly cloudy conditions using the Pocket Light Meter, Version 6 (Nuwaste Studios) iPhone application and converted from lux]. This difference in light availability and the border between the open meadow and wooded areas did not seem to hinder moth movements between the habitat types (as shown below in our results).

We acknowledge that our design (using one high density area and two adjacent low density areas) does not allow us to partition the effects of gross environmental differences among the areas from density effects. This is a constraint of conducting experimental manipulations where the environment naturally varies. Thus, we present our results in terms of area effects. However, our results (below) are consistent with a density effect mediating the interaction of *H. ectypa* with *S. stellata*. We can assert that area effects are consistent with density effects because we found that our two low density areas did not differ statistically at *P* < 0.05 for any of our measured variables (Appendix, Table A1).

### Experimental design

We used potted plants to quantify the effect of host plant density on pollinator visitation, pollen grain deposition onto stigmas, seed set, and *H. ectypa* oviposition and predation. Potted *S. stellata* plants were started from seeds haphazardly collected from plants growing within the large, continuous population (HDM) in 2006. Plants were grown in the greenhouse until July 2008 and from September 2008 through July 2009 with ambient temperature and light and watered as needed (with 4 months outdoors each winter). High density study plants were placed within the continuous established population (HDM; Fig. 1). For the low density context, we placed potted plants in the LDW and LDM areas at least 30 m from the nearest naturally occurring *S. stellata* plant because this distance is more than 10-fold greater than the mean (2.2 ± SE 0.43 m) interplant flight distances of moths visiting *S. stellata* (Reynolds et al. 2009; Fig. 1). In both years potted plants were placed in the field 11 July.

Once deployed in the field, potted plants were watered by hand if no rain had fallen in the previous 5 days or if the soil in the pot showed evidence of drying. Because all study plants were potted in the same soil and water moisture levels were maintained at similar levels at the greenhouse and in the field, belowground resources should not differ among plants. All potted plants were protected from deer herbivory by either individual cages with 2-inch chicken wire or netted enclosures when plants were arranged in patches for pollinator observations. Both these protection methods allow for natural pollination, oviposition and predation by insects, as well as natural fruit set and maturation.

### Pollinator visitation

To quantify the effect of host plant density on *H. ectypa* visitation, we observed patches of 5–12 potted plants within the high and low density areas (30 m from the nearest established *S. stellata* plants; Fig. 1 hatched squares) on most nights from 11–25 July 2008 (11 nights) and 13 July–4 August 2009 (19 nights). Occasionally weather (rain or cold temperature) precluded observations, and it was unlikely that pollinators visited on those nights (personal observation). Observations began after sunset (∼8:45 pm E.S.T.) and lasted up to 2 h, corresponding to the period when most moth activity occurs (Reynolds et al. 2009). Pollinator type (*H. ectypa* or moth co-pollinator) was recorded for each moth pollinator visit. Patches within high and low density areas were observed simultaneously. In 2008, infrared camcorders (Sony Digital Handycams, model TRV17) using the night vision setting supplemented personal observations in both the high and low density patches and were fixed on groups of 3–10 flowers on three plants per patch each night. For the low density patches in 2008, observation nights alternated between LDW (*N* = 6 nights) and LDM (*N* = 5 nights). In 2009, for the last six observation nights, observations in the low density areas alternated between habitats (LDW and LDM) with three observations in each area, and camcorders were used in the high density area for those six nights. In both years, total number of flowers in a patch was counted prior to the start of observations each night and ranged from 49–201 flowers per patch in 2008 and 7–50 flowers per patch in 2009. In 2009, there were fewer flowers per patch compared to 2008 because observations took place over a longer period of time during the season, until the last of the individual plants initiated flowering. Visitation rate was calculated from number of visits by a pollinator type divided by the number of flowers in the patch on an observation night divided by the length of the observation period (usually around 1 h and up to 2 h). Before analysis, the visitation rate of each moth type in each patch on each night was square-root transformed to meet the assumptions of ANOVA. We performed ANOVA on the main effects of area (HDM, LDW, LDM) and pollinator type and their interaction on the number of moths per flower per hour in each year. Because of differences between numbers of sampling nights and number of plants observed, each year was analyzed separately.

### Pollination

To determine if the moths were equally effective in transferring pollen independent of the host plant density context, we quantified stigmatic pollen loads of flowers that were also used for pollinator observations (denoted as hatched squares in Fig. 1) in 2008 in all three areas. After a single pollinator visit to a virgin female flower (first night as female) during evening pollinator observation periods, the type of visitor (*H. ectypa* or co-pollinator) was recorded, the flower was placed in a glass tube and samples were transported to the lab where the stigmas were removed then fixed and stained in glycerine jelly with fuchsin on a microscope slide (Beattie 1971). Stigmas from unvisited flowers (as observed during the observation period) were fixed and used as baseline controls. Stigmatic pollen loads were counted using a compound microscope at 40× magnification. Before analysis, stigmatic pollen loads were log_10_ +1 transformed (to meet assumptions of ANOVA). We performed ANOVA on the main effects of area (HDM, LDW, LDM) and visitation and their interaction on transformed pollen grains per flower.

### Seed set, oviposition and predation

Individually placed potted plants were used to determine the effects of environmental context on seed set per mature fruit, oviposition, and flower and fruit predation by larvae in 2009 (in 2008, the focus on pollen deposition precluded the examination of these traits). Eighteen individual potted plants were placed into each of the three areas along transects at 30 m intervals on 11–12 July 2009 (denoted as filled circles in Fig. 1). In the HDM area, two parallel 270 m transects of nine plants each were separated by 30 m, and these plants were an average of 1.1 and 1.9 m from their nearest and second nearest naturally occurring *S. stellata* neighbors. For the LDW areas, two 270 m transects were established, one on each side of the continuous HDM population, 30 m into the woods and away from naturally occurring *S. stellata* plants in the HDM. In the LDM three 180 m linear transects 30 m apart were established perpendicular to the road with six plants 30 m apart per transect in a lower part of the same power line cut where the HDM is located (Figs. 1, 2). This design within the LDM area resulted in plants being located increasingly farther from the established *S. stellata* population in the HDM, however, there was no statistically significant effect of this distance on seed set, oviposition or predation within the LDM area (data not shown). This arrangement of potted plants maximized similar replication levels within the three study areas while maintaining (at least) the spatial separation among plants that was required to test our hypothesis about the role of host plant density on pollinating seed predator interaction outcomes. Mean number of flowers produced by the potted plants did not differ among the three areas (data not shown).

On individual plants deployed in 2009, all flowers and fruits were collected ∼21 days following flowering when fruits were mature but prior to fruit dehiscence (3 August–9 September). Three LDW plants died prematurely, for a final sample size of 15 plants in that area. The following data for each plant were collected in the lab using a dissecting scope at 15× magnification when necessary: total number of flowers and fruits, number of mature fruits, number of seeds in mature fruits, number of egg cases on fruits or uneaten flowers, and whether a flower or fruit was eaten by *H. ectypa* larvae (= predation).

In order to calibrate the ability of moths to act as pollinators to high and low density plants, we also quantified seed set from plants where no pollinator visitation was allowed to occur (pollinator exclusion plants, hereafter PE). Twelve naturally occurring plants of similar size to our potted plants were caged with fine mesh screen and chicken wire to exclude pollinators and to prevent deer herbivory within the high density meadow field site. Light availability under the cages was 72% lower than the level of light available for uncaged plants (Caged: 966.5 *μ*mol·s^−1^·m^−2^, Uncaged: 3516.9 *μ*mol·s^−1^·m^−2^).

Seed set per mature fruit was calculated as the number of seeds per number of mature uneaten fruits per plant for HDM, LDW, LDM, and PE plants. Nineteen low density plants (LDM = 6, LDW = 13) did not produce any mature, uneaten fruits and were therefore eliminated from these seed set analyses (though they were used to determine oviposition and predation). Seed set values were log_10_ transformed to meet assumptions for ANOVA to test for differences among HDM, LDW, LDM, and PE plants.

Additionally, for plants in the HDM, LDW, and LDM areas, oviposition and predation were also calculated. For each plant, oviposition was calculated as the number of eggs per total number of fruits and/or uneaten flowers. Eggs were counted in the lab on fruits and uneaten flowers; eaten flowers were excluded from this calculation because when flowers were eaten, the ovary and any egg(s) deposited were completely consumed. Due to extreme nonnormality of the residuals, oviposition rate differences among the three areas (HDM, LDW, LDM) were analyzed with a Kruskal–Wallis nonparametric test (Proc NPAR1WAY, SAS).

For our purposes, predation included both florivory and frugivory because *H. ectypa* larvae consume flowers and fruits, and predation was calculated as the proportion of all flowers and fruits eaten per total number of uneaten and eaten flowers and fruits on a plant. Predation was square transformed to meet assumptions for ANOVA to test for differences between the three areas (HDM, LDW, LDM). We used a generalized linear model to compare the three areas for differences in number of plants with high predation (>95%). Ninety-five percent is one standard deviation above the overall mean predation rate.

### Statistical analyses

All statistical analyses were performed in SAS 9.2 (SAS Institute, Cary, NC, 2009). All ANOVA tests were performed using Proc GLM. When post hoc means separation tests were required, Tukey's HSD was employed (unless otherwise noted). Differences in the presence of >95% predation on plants in each area were tested using a generalized linear model in Proc GENMOD with a binomial probability distribution (presence/absence of ≥95% predation) with a logistic link function. We used contrasts with a sequential Bonferroni correction to test for pairwise differences in the highest predation rates between areas.

## Results

### Pollinator visitation

In 2008, there was a significant interaction between area and pollinator type (*H. ectypa* vs. co-pollinator; *F*_2,43_ = 6.58, *P* = 0.0035). The highest visitation by *H. ectypa* occurred in the HDM area, intermediate visitation by *H. ectypa* occurred in the LDW, followed by the lowest visitation of *H. ectypa* in the LDM (Table 1). Co-pollinator visitation was significantly lower than *H. ectypa* visitation to the HDM and LDW areas, but visitation was similar for *H. ectypa* and co-pollinators in the LDM area (Table 1).

**Table 1:** Untransformed means ± 1 SE for visitation rate (number of moths per flower per hour) of *Hadena ectypa* and co-pollinators in 2008 and 2009 in high density meadow (HDM), low density woods (LDW) and low density meadow (LDM) areas

	HDM	LDW	LDM
2008			
*H. ectypa*	0.234 ± 0.031	0.180 ± 0.026	0.081 ± 0.038
Co-pollinators	0.015 ± 0.005	0.021 ± 0.010	0.016 ± 0.002
2009			
*H. ectypa*	0.281 ± 0.074	0.429 ± 0.095	0.155 ± 0.048
Co-pollinators	0.134 ± 0.046	0.057 ± 0.021	0.067 ± 0.023

Visitation rate was calculated from number of visits by a pollinator type divided by the number of flowers in the patch on an observation night divided by the length of the observation period. Each area-night was considered a replicate (2008 N: HDM = 11, LDW = 6, LDM = 5; 2009 N: HDM = 19, LDW = 16, LDM = 16). Comparing within each year and area *H. ectypa* visited *Silene stellata* at a significantly higher frequency than moth co-pollinators at *P* < 0.05 except for the LDM in 2008. Additionally, in 2008, *H. ectypa* visitation was significantly higher in HDM as compared to the LDM and LDW areas.

In 2009, co-pollinator visitation across all three areas was similar and significantly lower compared to *H. ectypa* visitation (*F*_1,101_ = 14.56, *P* = 0.0002; Table 1). However, total pollinator visitation rate (combined *H. ectypa* and co-pollinators) to plants at all three areas was similar (*F*_2,101_ = 1.93, *P* = 0.1511). Thus, there was no interaction between pollinator type and location, in terms of visitation (*F*_2,101_ = 2.29, *P* = 0.1068).

### Pollen loads

Samples from a total of 102 flowers were collected (*N* = 76 HDM, *N* = 13 for each LDW and LDM). There was no effect of *H. ectypa* behavior (nectar feeding only vs. nectar feeding and ovipositing) on pollen deposition (see also Kula et al. 2013), and samples from these two types of visits were combined within HDM, LDW, and LDM samples for further analysis.

Due to low moth co-pollinator visitation rates, only four samples from moth co-pollinator visits (all from *Autographa precationis*, Noctuidae) were collected. These samples were not included in the analyses: HDM: *N* = 1, 23 pollen grains; LDM: *N* = 3, mean = 9.33 pollen grains per flower. These values fall within the range of stigmatic pollen loads resulting from *H. ectypa* visits to plants in the high and low density areas, respectively (see below).

**Figure 3:**
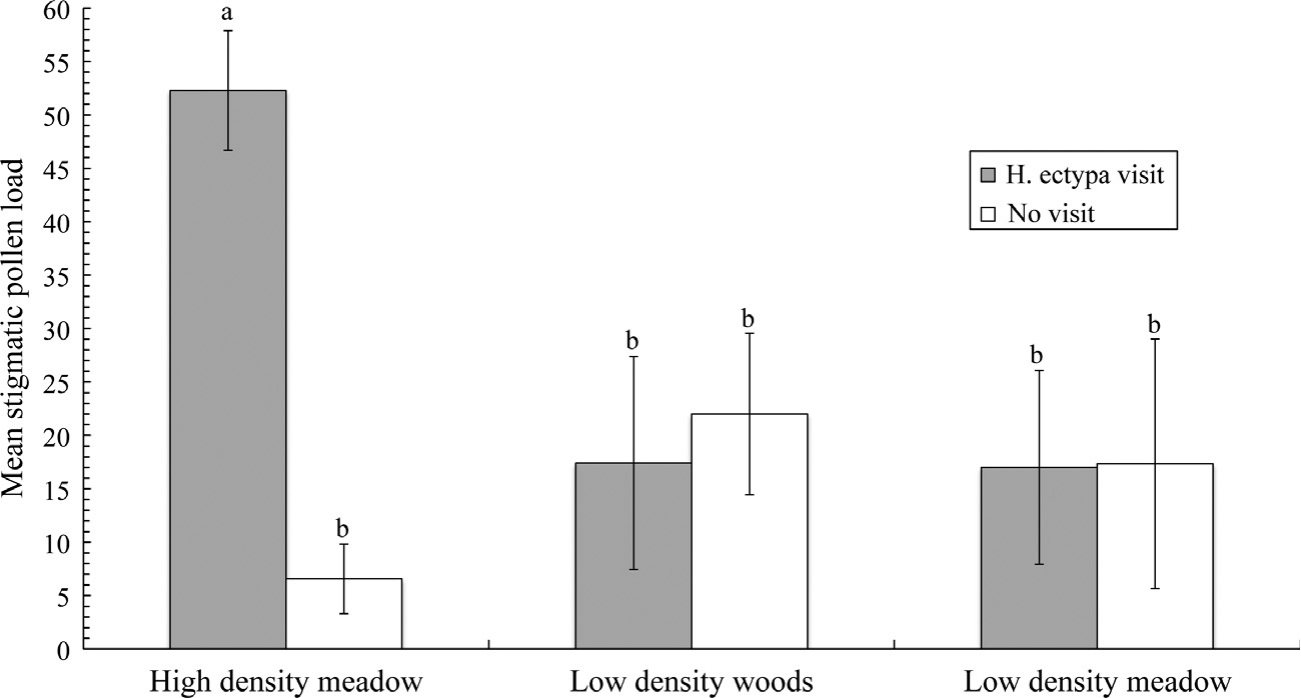
Stigmatic pollen loads from flowers collected from individual potted plants in the high density (HDM) and low density areas (LDW and LDM) in 2008. The means and standard errors presented are untransformed, however, statistical analyses were performed on log_10_ transformed data. Error bars represent ± 1 SE. Different letters above the bars represent significant differences between transformed means. Average ovule production per flower of *Silene stellata* is 25 (Reynolds et al. 2009).

There was a significant interaction between visitation (visit by *H. ectypa* vs. no visit) and area on stigmatic pollen loads that is consistent with density effects (*F*_2,96_ = 6.71, *P* = 0.0019; Fig. 3). In the HDM area, *H. ectypa* stigmatic pollen loads were more than double the loads found in both the low density areas or on unvisited high density flowers. In the low density areas, stigmatic pollen loads were not statistically different between flowers visited by *H. ectypa* and unvisited flowers. As expected, stigmatic pollen loads on unvisited flowers did not differ among the three areas. Main effects were not significant (area: *F*_2,96_ = 0.38, *P* = 0.6863; visitation: *F*_1,96_ = 0.02, *P* = 0.8765).

### Seed set, oviposition, and predation

Of the plants that produced at least one mature uneaten fruit, seed set per mature fruit was highest for plants in the HDM area, twice as great when compared to LDM and PE plants. LDW plants had the lowest seed set (Table 2, *F*_3,41_ = 4.59, *P* = 0.0074).

**Table 2:** Untransformed means ± 1 SE for plant reproductive measures taken on individually potted plants placed in the high density meadow (HDM, *N* = 18), low density woods (LDW, *N* = 15) and low density meadow (LDM, *N* = 18) areas in 2009

	HDM	LDW	LDM	PE
Seeds per fruit	14.5 ± 2.22	7.75 ± 6.79	6.80 ± 1.48	6.54 ± 1.49
Oviposition	0.152 ± 0.040	0.078 ± 0.048	0.173 ± 0.60	NA
Predation	0.544 ± 0.054	0.652 ± 0.084	0.668 ± 0.074	NA

Oviposition was calculated as the number of eggs per number of fruits or uneaten flowers, and predation was calculated as the proportion of all flowers and fruits eaten per plant. Oviposition and predation were not examined on pollinator exclusion (PE; *N* = 12) plants because those plants were not exposed to *H. ectypa* visitation, thus in the table these data are marked as not applicable (NA). Seed set per mature uneaten fruit (but not oviposition and predation) was significantly different among plants in the different groups.

There was no statistical differences in oviposition on plants among the three areas (Table 2, 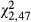 = 4.8523, *P* = 0.0884). Approximately one out of every ten flowers on an individual plant received an egg independent of its location (i.e., plant density) within the study site.

Mean predation of flowers and fruits per plant did not differ significantly among the three host plant areas (Table 2, *F*_2,48_ = 2.03, *P* = 0.1429), with a mean of over 50% of flowers and fruits eaten on plants in each area. However, the number of plants with >95% predation differed among areas (*χ*^2^ = 7.856, *P* = 0.0198). The two low density areas had a similar number of high predation plants (*χ*^2^ = 0.09, *P* = 0.7670), but both LDW (*χ*^2^ = 6.98, *P* = 0.0082) and LDM (*χ*^2^ = 6.05, *P* = 0.0139) had more plants with predation over 95% compared to the HDM (*P* < 0.05 following sequential Bonferroni correction).

## Discussion

We found that female reproductive success for *S. stellata*, due to facultative interactions with its specialized pollinating seed predator, was significantly reduced based on the environmental context of the interaction (demonstrating context dependency in the interaction outcome). While we recognize the potential for effects of host plant density to be confounded by additional differences among the study areas (e.g., light levels, surrounding plant community), especially between the two meadow areas compared to the wooded area, our results are congruent with an effect of *S. stellata* density as a mediator of individual interactions with *H. ectypa*. First, for this same study system, Reynolds et al. (2012) showed that oviposition rate was not significantly different for naturally occurring plants in our high density meadow and a nearby (but not adjacent) wooded area with lower natural density (personal observation), a result that agrees with our study's findings. Further, in testing for an effect of habitat type (analyses not shown) at low density, we found that our two low density areas did not differ statistically at *P* < 0.05 for any of our measured variables (Appendix, Table A1). Finally and most importantly, we quantified a mechanism for density dependent effects that should be independent of other gross environmental differences between the sites. In the low density areas, *H. ectypa* transferred pollen at a much lower rate than within the high density areas, consistent with expectations of low pollen dispersal distances (see below for further discussion). Consequently, these results make a compelling argument for our experimental design employing density manipulations across different environmental settings within the population as an acceptable approach for comparing interaction outcomes for host plant reproduction among different environmental contexts.

Because of higher *H. ectypa* visitation rates compared to moth co-pollinators and equivalent pollinator effectiveness (stigmatic pollen loads) of *H. ectypa* and co-pollinators (Reynolds et al. 2012), *H. ectypa* was the most important pollinator for both high and low density areas. Additionally, in 2008, *H. ectypa* visitation was significantly greater in the HDM versus the low density areas. Lower *H. ectypa* pollinator effectiveness in the low density areas (as compared to HDM) likely resulted in the lower seed set we found for host plants at low density. Oviposition was similar among the areas, and predation was highest in the low density areas. These combined results indicate *H. ectypa* has a greater negative effect on *S. stellata* female reproductive success in the low density areas, as predicted. We show that the primary mechanisms driving differences in interaction outcomes at high and low host plant densities is context-dependent pollinator efficiency (pollen transfer rates), subsequent seed set and predation. When plants occur at low density, as *S. stellata* frequently does (personal observations), the interaction outcome is clearly antagonistic for the host plant, thereby demonstrating the potential for this pollinating seed predator interaction to limit population establishment or population maintenance during range expansion or following habitat fragmentation.

Several previous pollinating seed predator studies have documented context-dependent differences in interaction outcomes resulting from variable plant population size or density while using a diversity of approaches ranging from experimental versus survey and focusing on either the plant or insect partner (Holland and Fleming 1999; Elzinga et al. 2005; Despres et al. 2007; Klank et al. 2010; Reynolds et al. 2012). We add to this literature by examining the effect of host plant density on the pollination efficiency of a pollinating seed predator at the within-population level in addition to the resulting seed set and predation, which others have not explored.

We predicted that in low density areas, co-pollinator visitation would be lower than *H. ectypa* visitation, and overall our results from both years confirmed that prediction. In fact, aside from HDM in 2008 when co-pollinator and *H. ectypa* visitation were similar, we found consistently low co-pollinator visitation to plants within all areas, which may be indicative of low co-pollinator abundance across the entire population. The relatively higher co-pollinator visitation to flowers in 2009 is likely due to the extension of visitation observations into the latter half of the season when co-pollinators are known to be consistently more abundant in the population (Kula 2012; Reynolds et al. 2012). Our results (1) are similar to results from another moth pollinating seed predator system with co-pollinators (*Lithophragma*–*Greya* system, Cuautle and Thompson 2010) and (2) demonstrate that co-pollinators were not reliable pollinators of *S. stellata* regardless of host plant density, especially early in the flowering season when these studies were conducted and when co-pollinators are consistently less abundant (Kula 2012; Reynolds et al. 2012).

Indeed, the temporal dynamics of this study system with interannual differences in both flowering phenology and pollinating seed predator and co-pollinator relative abundance (both within and across flowering seasons; Kula 2012; Reynolds et al. 2012) may have implications for long-term ecological outcomes and evolutionary persistence of the pollinating seed predator interaction, especially in the context of climate change effects on biotic interactions (Blois et al. 2013). These questions are being addressed by our research group (Kula 2012, A. A. R. Kula et al. in prep.) and will shed light on how changing phenology affects synchrony of host plant flowering and *H. ectypa* adult activity (pollination and oviposition) and the outcome of the interaction for host plant reproduction.

Further, *H. ectypa* visitation response to host plant density differed between years. In 2008, *H. ectypa* visitation to HDM plants was greater than to plants in the low density areas, which is in contrast to 2009, when there was no difference in *H. ectypa* visitation among the three areas. These results are especially surprising considering patch sizes in 2009 were smaller than in 2008 but also indicate that in some years *H. ectypa* may be able to find *S. stellata* regardless of host plant density.

*Hadena ectypa* transferred significantly smaller stigmatic pollen loads to plants residing within both low density contexts versus plants in the high density area. Stigmatic pollen load differences likely were driven by (1) lower pollen availability (fewer pollen donors) in low density areas (Moeller 2004; Knight et al. 2005) and (2) less pollen carryover to the low density areas from the nearby continuous HDM. Insect-mediated pollen dispersal distances of tens of meters have been reported for *Silene latifolia* (as *Silene alba*) (McCauley 1997; Richards et al. 1999). However, nocturnal moth pollinators of *S. stellata* moved fluorescent dye (as a pollen analog) only 2.2 m on average (Reynolds et al. 2009), and pollen has been found to be quickly removed from the proboscis of *Hadena bicruris* (Brantjes 1976). Thus, it is not surprising that *H. ectypa* moths were inefficient pollinators of *S. stellata* when traveling 30 m from a high density area of plants to a low density area. It is also possible that *H. ectypa* adults flew to low density areas and repeatedly visited the same flowers, quickly depleting the limited supply of pollen (Schulke and Waser 2001). Regardless of the mechanism, the low stigmatic pollen load at low plant density resulting after a *H. ectypa* visit was a major factor leading to an antagonistic outcome of this pollinating seed predator interaction for the host plant in this environmental context. Therefore, stigmatic pollen load is an important, although understudied, variable for studies of density dependent variation in outcomes of pollinating seed predator interactions.

We propose that seed set differences for plants in high versus low density areas are attributable to pollen limitation rather than resource limitation because fruit initiation was consistent throughout the entire flowering season. This pattern and reasoning was also noted by Knight et al. (2005), although we do not have experimental confirmation of pollen limitation of seed set. In low density areas, the similar stigmatic pollen loads of unvisited and visited flowers indicate that pollinator-mediated pollen deposition likely made only small (if any) contributions to seed set in low density areas, with most seeds likely the result of self-pollination. Consistent with this pattern, seed set of low density and pollinator exclusion plants were similar. Self-pollination, however, is often detrimental to plant fitness because it may result in inbreeding depression and have important consequence on seed number and quality (e.g., Dudash 1990; Collin et al. 2009). If plants at low density primarily produce seed from self-pollen, this phenomenon could indicate one more mechanism by which low host plant density results in negative outcomes of pollinating seed predator interactions for the host plant in this context.

Oviposition was similar for plants in high and low density areas, corresponding to the similar visitation rates of *H. ectypa* to these areas in 2009, as oviposition is a reliable proxy for *H. ectypa* visitation (Reynolds et al. 2012). These results also are in agreement with the findings reported by Reynolds et al. (2012) of similar oviposition rates on naturally occurring plants in the HDM and a nearby wooded area (similar in habitat to our LDW area but with an established *S. stellata* population that is lower in density than the HDM). In contrast to our results, Elzinga et al. (2005) found higher oviposition in isolated populations (100 m away), and the authors suggested that in low density patches, pollinating seed predators may choose to remain and thereby lay more eggs. Together our results suggest that there could be a threshold effect for pollinating seed predator behavioral responses to density and distance: beyond a certain density and distance (determined at least partly by mobility of the insect), responses may change (Groom 2001).

Mean flower and fruit predation did not differ among high and low density areas. However, we observed that both LDW and LDM had more plants at the highest levels of predation (≥95%) compared to the HDM, which was consistent with our prediction for higher likelihood of predation in the low density areas. There are multiple possible mechanisms to explain this pattern. First, as we predicted, larvae on low density plants may have consumed more of the hardened, less favorable fruits because no other suitable plant material was available (Kunin 1997; Castillo et al. 2013). Second, higher natural enemy (e.g., parasitoid) abundance is expected at higher plant density, such that low host plant density areas are enemy-free spaces (Jeffries and Lawton 1984), resulting in elevated predation at low density due to increased larval survival.

Others have shown variation in the outcome of plant–insect interactions among populations that span across broad spatial scales and environmental gradients (Louda 1982; Rand 2002; Miller et al. 2009) and the potential for geographic mosaics of coevolution between insects and plants (Thompson and Cunningham 2002; Thompson and Fernandez 2006; Thompson et al. 2010; Reynolds et al. 2012). However, our study focuses on interactions at a relatively small spatial scale (tens of meters) and demonstrates that varying ecological context even within a population can affect the outcomes of a potential mutualism. For pollinating seed predator interactions, in particular, no other study has fully demonstrated this phenomenon by examining pollinator visitation rates and pollen grain deposition (= pollinator importance, Reynolds et al. 2009) along with seed set, oviposition and predation for high density and low density areas within a population. Our findings that the detrimental effects of pollinating seed predators on host plant reproductive success is less in high density areas compared to low density areas contributes to our understanding of the role of ecological context in the persistence of *Silene*–*Hadena* interactions despite the presence of co-pollinator mutualists (Kephart et al. 2006; Bernasconi et al. 2009) and perhaps to other facultative pollinating seed predator host plant associations by revealing a potential pathway between parasitism, mutualism and coevolution (Dufay and Anstett 2003; Thompson and Fernandez 2006).

## Appendix

**Table A1:** Statistical results (*F* statistics, degrees of freedom and *P* values) for ANOVA comparisons between the low density woods and low density meadow areas for all response variables examined

	*F*	df	*P*
2008 visitation	1.28	1,20	0.2719
2009 visitation	3.04	1,62	0.0864
Pollen deposition	0.02	1,24	0.8797
Seed set	1.75	1,14	0.2067
Oviposition	1.59	1,29	0.2169
Predation	0.02	1,31	0.8921
